# A Randomized Control Trial of Cognitive Compensatory Training (CCT) and Computerized Interactive Remediation of Cognition-Training for Schizophrenia (CIRCuiTS)

**DOI:** 10.3389/fpsyt.2022.878429

**Published:** 2022-07-01

**Authors:** Frances Louise Dark, Victoria Gore-Jones, Ellie Newman, Maddison Wheeler, Veronica Demonte, Korinne Northwood

**Affiliations:** ^1^Metro South Addiction and Mental Health Services, Brisbane, QLD, Australia; ^2^School of Medicine, The University of Queensland, Brisbane, QLD, Australia

**Keywords:** schizophrenia, cognitive remediation, cognitive compensation, randomized controlled trial, cognitive rehabilitation

## Abstract

**Background:**

Various modes of delivering cognitive remediation (CR) are effective, but there have been few head-to-head trials of different approaches. This trial aimed to evaluate the relative effectiveness of two different programmes, Cognitive Compensatory Training (CCT) and Computerized Interactive Remediation of Cognition—Training for Schizophrenia (CIRCuiTs).

**Methods:**

The study used a single-blind randomized, controlled trial to examine the efficacy and effectiveness of the two therapies. The study aimed to enroll 100 clinically stable patients between the ages of 18 and 65 years who had been diagnosed with a schizophrenia spectrum disorder. Participants were randomized to either the CCT or CIRCuiTs therapy groups. The primary outcome measures were neurocognition using the Brief Assessment of Cognition Scale (BACS) and the Subjective Scale to Investigate Cognition in Schizophrenia (SSTICS). The secondary measure was functional outcomes using the Social Functioning Scale (SFS).

**Results:**

There was no group difference in any of the outcome measures post-intervention or at follow-up. Both groups had a small improvement on their SSTICS scores between baseline (M = 30.52 and SD = 14.61) and post-intervention (M = 23.96 and SD = 10.92). Verbal memory scores as measured by list learning improved for both groups between baseline (*z* = −1.62) and 3-month follow-up (*z* = −1.03). Both groups improved on the token motor task between baseline (*z* = −1.38) and post-intervention (*z* = −0.69). Both groups had a decline in Symbol Coding scores between baseline (z = 0.05) and 3-month follow-up (z = −0.82).

**Discussion:**

This underpowered study found no difference in effect between the two approaches studied. If future studies confirm this finding, then it has implications for services where cost and lack of computer technology could pose a barrier in addressing the cognitive domain of schizophrenia spectrum disorders. The final sample size compromised the power of the study to conclusively determine a significant effect.

## Introduction

Cognitive deficits in schizophrenia spectrum disorders are common and are linked to poor functional outcomes. Cognitive remediation (CR) is defined as “an intervention targeting cognitive deficits using scientific principles of learning with the goal of improving functional outcomes. Its effectiveness is enhanced when provided in a context (formal or informal) that provides support and opportunity for extending everyday functioning” (1) is increasingly recommended in clinical practice guidelines for schizophrenia spectrum disorders, with services having to decide on how to implement CR in heterogenous routine care settings (2). Evidence is still being developed for CR on who responds to what, when, and how. Meta-analysis indicates various approaches to CR can be effective in improving neurocognitive abilities, such as attention, working memory, cognitive flexibility, planning, and executive functioning (3, 4). There is also evidence that functional improvement occurs when CR is combined with rehabilitation interventions (3).

Studies examining the relative effectiveness of different approaches to addressing the cognitive impact of psychosis are needed to assist services in selecting a CR approach that best fits their organization and is congruent with not only the individual but also the broader socioeconomic cultural context (5). Kidd et al. compared a compensatory cognitive approach with a restorative approach in an early psychosis population in Canada (6). Both interventions demonstrated significant effects on community functioning. In the developed world, where access and cost of computers are less of a barrier to care, CR is commonly delivered *via* computers-based programs (3). In other settings, especially low- and middle-income countries, infrastructure capability and cost can be major factors in implementing CR (5). Pen and paper programs to address cognitive deficits have been previously developed and evaluated (3, 7, 8).

### Objectives

The study aimed to explore the non-inferiority of Cognitive Compensatory Training (CCT) compared with Computerized Interactive Remediation of Cognition—Training for Schizophrenia **(**CIRCuiTs), primarily on measures of neurocognition and secondarily on measures of functioning.

Participants were community-based and registered with a public mental health service. All participants in the study continued to receive standard clinical care (i.e., there were no restrictions on medication or psychosocial interventions, apart from participants receiving therapies addressing neurocognition). The two interventions were delivered by trained mental health staff once (CCT, 2-h session) or two times (CIRCuiTs, 1-h sessions) per week for 12 weeks. Groups were based on a maximum of 6 participants per facilitator. Individual clinical assessments were conducted at baseline, post-treatment, and at three-month follow-up.

### Design

The design was a single-blind randomized control trial. Participants were randomized in a 1:1 ratio to join either the CCT or CIRCuiTS group, using a computer-generated randomization table. The power analysis was based on the primary outcome measure, the Brief Assessment of Cognition Scale (BACS). An attrition rate of 15–20% was estimated giving a proposed final sample size of approximately 80 participants. The attrition rate was based on our outpatient pilot study, which had a retention rate of 83%, as well as our previous experience with group psychosocial randomized controlled trials research (9).

The primary outcomes were cognitive tests (BACS and Subjective Scale to Investigate Cognition in Schizophrenia [SSTICS]) administered at baseline, post-treatment, and 3-month follow-up. Secondary outcomes, the Social Functioning Scale (SFS) and the Brief Psychiatric Rating Scale (BPRS) were also measured at the three time points. Assessments were administered by trained research assistants and clinical psychologists who were blind to the condition.

## Method

### Study Setting

The study was conducted in a Community Mental Health Center.

### Eligibility Criteria

The specified inclusion criteria were: (1) aged between 18 and 65 years (inclusive), (2) fulfilling the clinical diagnosis of DSM-V criteria for schizophrenia spectrum disorder, (3) the absence of uncorrected sensory impairments, (4) English literacy skills greater than grade 4 as per years of education, and (5) agreement to participate, with the capacity to consent and able to follow the study instructions and procedures.

The specific exclusion criteria were: (1) the presence of substance dependence (except for tobacco), (2) intellectual disability (estimated full-scale IQ less than 70), (3) people who were unable to understand or communicate in English or with English literacy skills of less than grade 4 as per years of education, and (4) comorbid physical illnesses that would impair the participants' ability to complete the trial.

### Interventions

#### Cognitive Compensatory Training

Cognitive Compensatory Training is a 2-h × 12 session manualised CCT program that focuses on the use of strategies to improve real-world cognitive functioning. CCT is delivered through paper-and-pen tasks, usually in a group format (10). The program focuses on the domains of prospective memory, attention, and vigilance, learning and memory, and executive functioning *via* the use of strategies, such as use of calendar, verbalizing while completing tasks, note-taking, and a six-step problem-solving method. Eleven completed sessions were considered an adequate treatment exposure.

#### Computerized Interactive Remediation of Cognition—Training for Schizophrenia

Computerized Interactive Remediation of Cognition—Training for Schizophrenia is a computer program that includes tasks for a wide range of cognitive functions (particularly executive function and memory). This program has a focus on metacognition as well as strategic processing and drill and practice (11). The cognitive tasks are set in a simulated village with activities undertaken in the simulated context in which the tasks may need to be used in real life, such as planning a shopping trip in a supermarket. Metacognition is targeted by getting the participant to choose the strategies they will need for a particular task, estimating the time required to complete the task, and then rating the usefulness of the strategies and actual time taken following task completion and immediate feedback. In this study, CIRCuiTs ran for 1 h two times a week with a trained therapist in a group of up to 6 participants. CIRCuiTS consist of 40 stages. Twenty sessions were considered adequate treatment exposure.

### Outcome Measures

A battery of validated clinical measures was conducted at baseline, post-treatment, and at 3-month follow-up. Raters who were blind to the randomization of conditions completed the measures.

The following measures were used:

1. The Weschler Test of Adult Reading, the Test of Premorbid Functioning (TOPF) (12) is a reading test estimating premorbid intelligence as estimated from reading ability. It takes approximately 10 min and is composed of a list of 70 words. It was used to screen for intellectual disability.
2. Brief Assessment of Cognition Scale (BACS) is an instrument that assesses five domains of cognition with six tests taking approximately 30 min (13). The six tests are list learning (verbal memory); digit sequencing (working memory); token motor task (motor speed); verbal fluency (semantic fluency and letter fluency); Tower of London (reasoning and problem solving); and Symbol Coding (attention and processing speed). Equivalent forms are available.
3. Subjective Scale to Investigate Cognition in Schizophrenia (SSTICS) (14). This 21-item Likert self-report scale-based measure assesses the subjective perception of functional cognitive abilities when completing everyday tasks as rated by the individual. Each item is rated based on a scale from 0 never to 4 very often, with a higher score reflecting more subjective cognitive impairment. The items assess the domains of sustained executive function, memory for information, the consciousness of effort, cognition in daily life, distractibility, and alertness. It has been found to have good internal consistency (alpha = 0.88) and stability over time (14).
4. Social functioning Scale (SFS) assesses areas of functioning that are crucial to the community maintenance of individuals with schizophrenia (15). It is a self-reported measure of 79 items representing seven dimensions: social engagement, interpersonal behavior; pro-social activities; recreational activities; independence-competence; independence-performance, and employment. A higher score represents better functioning.
5. Brief Psychiatric Rating Scale (BPRS) (16, 17) is a widely used scale for measuring symptoms of patients in mental health and has been extensively used in studies of patients with schizophrenia. Symptom severity is rated from 1, not present to 7 extremely severe. High scores represent greater symptom severity.

### Participants

Participants were community-based and registered with a public mental health service. All participants in the study continued to receive standard clinical care (i.e., there were no restrictions on medication or psychosocial interventions, apart from participants receiving therapies addressing neurocognition).

**Figure 1:**
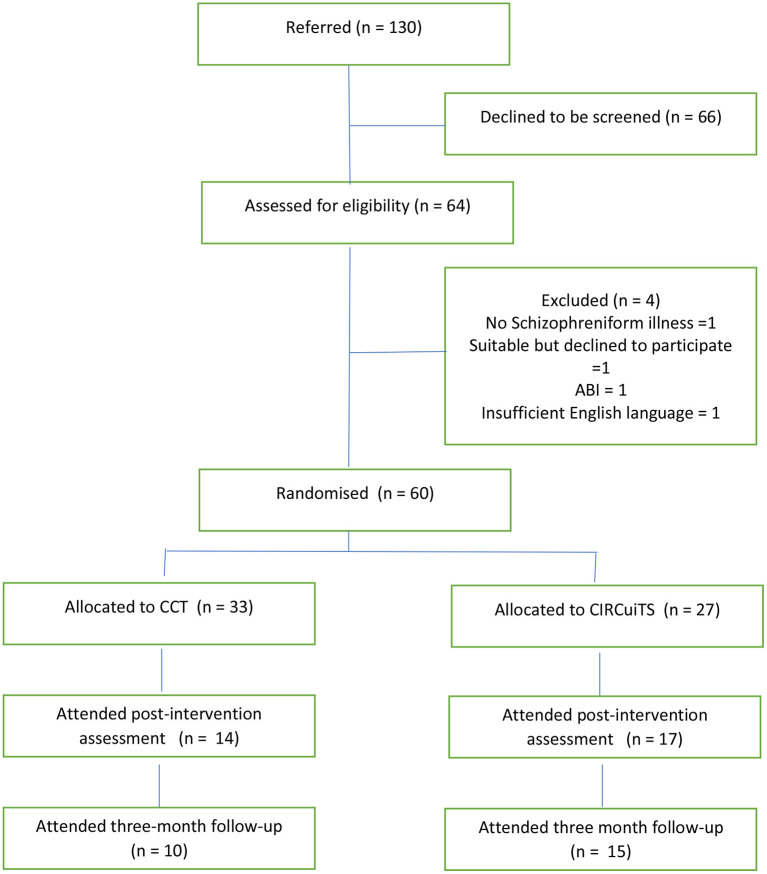
CONSORT flow diagram.

At the time the study had to be canceled, one hundred and thirty mental health outpatients were screened for eligibility. Furthermore, sixty-six declined to be screened and sixty-four were assessed for eligibility (Figure 1). Sixty participants who met the criteria for a schizophrenia spectrum disorder (DSM-V) were randomized to either the CCT or CIRCuiTS groups. Participants were between 18 and 65 years of age and did not have an intellectual impairment based on their Test of Premorbid Functioning scores.

### Analyses

Brief Assessment of Cognition Scale scores were converted to *z*-scores prior to the analyses. The primary efficacy analysis assessed average treatment group differences for the primary outcome measure BACS and SSTICS, over the entire study period (baseline, post-intervention, and follow-up), and used a series of 2 (group: CCT and CIRCuiTS) × 3 (time: baseline, post-intervention, and 3-month follow-up). Mixed factorial ANOVAs with repeated measures on the second factor were conducted on the primary (BACS subtests, SSTICS) and secondary outcome measures (SFS subtests and BPRS). This analysis used the Statistical Package for Social Sciences (SPSS Version 27). The statistical significance was set at *p* < 0.01.

## Results

The groups did not differ significantly on baseline demographic and cognitive variables (as shown in Table 1). There was no impact of other confounding variables (such as age, gender, premorbid IQ, or Olanzapine-equivalent medication dosage) on the outcomes.

**Table 1:** Means and standard deviations (SD) for baseline demographic and cognitive variables.

**Variable**	**Circuits (*n =* 15)**	**CCT (*n =* 10)**	**Total (*n =* 25)**	**Test statistic, *p-*value**
Age (in years)	33.53 (9.73)	32.50 (8.06)	33.12 (8.94)	*t* = −0.28, *p* = 0.78
Males no. (%)	12 (80)	9 (90)	21 (84.00)	χ^2^ = 0.45, *p* = 0.50
Olanzapine-equivalent medication dose	34.72 (31.63)	32.83 (32.57)	33.96 (31.34)	t = −0.15, *p* = 0.89
SSTICS	32.20 (14.65)	28.00 (14.95)	30.52 (14.61)	*t* = −0.70, *p* = 0.49
Social functioning scale	127.68 (7.22)	123.87 (9.25)	126.16 (8.13)	*t* = −1.15, *p* = 0.26
BPRS	35.93 (8.07)	38.00 (12.66)	36.76 (9.96)	*t* = 0.50, *p* = 0.62
**BACS**				
A. Composite B. Verbal memory C. Digit span D. Verbal fluency E. Symbol coding F. Tower of London G. Token motor	−1.98 (2.80) −1.49 (1.28) −1.26 (0.85) −0.89 (1.04) −0.97 (0.99) −0.06 (0.81) −1.24 (1.15)	−1.57 (0.72) −1.82 (0.72) −1.18 (0.40) −0.90 (1.37) −1.53 (0.62) 0.22 (0.98) −1.58 (0.63)	−1.82 (2.19) −1.62 (1.08) −1.23 (0.69) −0.90 (1.15) −1.20 (0.89) 0.05 (0.87) −1.38 (0.98)	*t* = 1.42, *p* = 0.16 *t* = −0.75, *p* = 0.46 *t* = 0.29, *p* = 0.77 *t* = −0.02, *p* = 0.99 *t* = −1.59, *p* = 0.12 *t* = 0.78, *p* = 0.44 *t* = −0.33, *p* = 0.75

*p = 0.05. SSTICS, Subjective Scale to Investigate Cognition in Schizophrenia; BPRS, Brief Psychiatric Rating Scale; BACS, Brief Assessment of Cognition in Schizophrenia*.

Twenty-nine (48%) participants failed to complete the programs (19, 57% CCT, 10, 37% CIRCuiTs). Eleven (9 CCT, 2 CIRCiuTs) were unavailable for a 3-month follow up (Figure 1).

### Brief Assessment of Cognition Scale

There was no significant main effect of group or group × time interaction effect on the BACS composite score (as shown in Table 2). There was a significant large main effect of Time on BACS composite scores (*p* = 0.04 η^2^ = 0.16) (Table 2). Composite scores improved for both groups between baseline (*z* = −1.82) and post-intervention (*z* = −0.96), and between baseline and 3-month follow up (*z* = −0.86) (Table 2).

**Table 2:** Mixed ANOVA results for main effect of time.

	**Baseline**			**Post-Intervention**			**3-month follow-up**			**Group X time interaction**			**Time main effect**			**Group main effect**		
**Measure**	**Circuits** **(*n =* 15)**	**CCT** **(*n =* 10)**	**Total** **(*n =* 25)**	**Circuits** **(*n =* 15)**	**CCT** **(*n =* 10)**	**Total** **(*n =* 25)**	**Circuits** **(*n =* 15)**	**CCT** **(*n =* 10)**	**Total** **(*n =* 25)**	**F**	***P* value**	**η^2^**	**F**	***P* value**	**η^2^**	**F**	***P* value**	**η^2^**
SSTICS	32.20 (14.65)	28.00 (14.95)	30.52 (14.61)	26.24 (10.99)	22.40 (12.83)	23.96 (10.92)	26.20 (10.98)	23.10 (8.88)	24.96 (10.11)	0.20	0.82	0.01	6.26	0.003**	0.21	0.46	0.50	0.02
SFS	127.68 (7.22)	123.87 (9.25)	126.16 (8.13)	130.42 (10.34)	125.86 (7.49)	128.59 (9.41)	127.01 (10.73)	122.26 (11.41)	125.11 (11.03)	0.10	0.91	0.00	2.92	0.91	0.00	1.81	0.19	0.07
BPRS	35.93 (8.07)	38.00 (12.66)	36.76 (9.96)	28.24 (5.75)	28.67 (9.54)	30.16 (7.58)	30.87 (8.03)	32.30 (10.42)	31.44 (8.88)	0.19	0.83	0.01	7.56	0.001**	0.25	1.81	0.19	0.07
**BACS**										
A. Composite B. Verbal memory C. Digit span D. Verbal fluency E. Symbol coding F. Tower of London G. Token motor	−1.98 (2.80) −1.49 (1.28) −1.26 (0.85) −0.89 (1.04) −0.97 (0.99) −0.06 (0.81) −1.24 (1.15) −1.24 (1.15)	−1.57 (0.72) −1.82 (0.72) −1.18 (0.40) −0.90 (1.37) −1.53 (0.62) 0.22 (0.98) −1.58 (0.63) −1.58 (0.63)	−1.82 (2.19) −1.62 (1.08) −1.23 (0.69) −0.90 (1.15) −1.20 (0.89) 0.05 (0.87) −1.38 (0.98) −1.38 (0.98)	−0.76 (0.90) −0.89 (0.90) −0.78 (1.05) −0.75 (1.18) −0.75 (1.13) 0.27 (1.38) −0.37 (1.11)	−1.27 (0.88) −1.04 (1.38) −0.94 (1.02) −0.42 (1.28) −0.81 (1.02) 0.32 (8.56) −1.18 (0.99)	−0.96 (0.91) −1.14 (0.96) −1.03 (0.94) −0.64 (1.10) −0.99 (0.99) 0.21 (1.27) −0.69 (1.12)	−0.61 (0.94) −0.95 (1.02) −0.94 (1.20) −0.51 (1.14) −0.59 (0.99) 0.53 (0.79) −0.52 (0.95)	−1.24 (0.54) −1.14 (1.04) −1.41 (0.70) −0.73 (1.44) −1.17 (0.62) −0.01 (0.84) −0.52 (0.95)	−0.86 (0.85) −1.03 (1.01) −1.13 (1.04) −0.60 (1.24) −0.82 (0.90) 0.32 (0.84) −0.69 (0.80)	1.59 0.74 2.40 2.40 0.13 2.47 1.00	0.22 0.48 0.10 0.10 0.88 0.09 0.38	0.07 0.03 0.09 0.08 0.00 0.09 0.04	4.35 5.07 0.88 0.88 4.78 0.53 9.14	0.04* 0.01** 0.42 0.42 0.01** 0.59 0.001**	0.16 0.17 0.04 0.04 0.17 0.02 0.28	0.26 1.67 0.76 0.01 3.11 0.15 2.54	0.61 0.21 0.39 0.91 0.09 0.70 0.13	0.01 0.07 0.03 0.00 0.11 0.00 0.10

*p < 0.05. **p < 0.01. SSTICS, Subjective Scale to Investigate Cognition in Schizophrenia; SFS, Social Functioning Scale; BPRS, Brief Psychiatric Rating Scale; BACS, Brief Assessment of Cognition in Schizophrenia.

There were no significant main effects or interaction effects on verbal fluency, digit span, or the Tower of London subtest. There was a large, significant main effect of time on list learning (verbal memory) scores (*p* = 0.01, η^2^ = 0.17). Verbal memory scores as measured by list learning improved for both groups between baseline (*z* = −1.62) and 3-month follow-up (*z* = −1.03). There was no significant difference between baseline and post-intervention.

There was no significant main effect of group or group × time interaction on the symbol coding subtest. However, there was a large significant main effect (*p* = 0.01, η^2^ = 0.17) of time between baseline (*z* = 0.05) and 3-month follow-up (*z* = −0.82). There was no significant difference between baseline and post-intervention (*z* = −0.99).

There was no significant main effect of group or group × time interaction on the token motor task. There was a large significant main effect of time (*p* = 0.001, η^2^ = 0.28) between baseline (*z* = −1.38) and post-intervention (*z* = −0.69). There was also a large effect of time between baseline and 3-month follow-up (*z* = −0.69).

### Subjective Scale to Investigate Cognition in Schizophrenia

There was no significant main effect of group or group × time interaction effect on SSTICS scores (as shown in Table 2). There was a large significant main effect of time for both ANOVA (*p* = 0.003, η^2^ = 0.21). Both groups had an improvement on their SSTICS scores between baseline (M = 30.52 and SD = 14.61) and post-intervention (M = 23.96 and SD = 10.92). There was no significant difference between baseline and 3-month follow-up (M = 24.96 and SD = 10.11).

### Secondary Measures

#### Brief Psychiatric Rating Scale

There was no significant main effect of group or group × time interaction effect on BPRS scores. There was a large significant main effect of time on scores (*p* = 0.003, η^2^ = 0.21). Both groups improved between baseline (M = 36.76 and SD =9.96) and post-intervention (M = 30.16 and SD = 7.58). There was a large statistically significant improvement between baseline and 3-month follow-up (M = 31.44 and SD = 8.88) (Table 2).

#### Social Functioning Scale

There were no significant main effects or group × time interaction effects on the SFS.

## Discussion

Cognitive remediation has been demonstrated to improve neurocognitive functioning in people with schizophrenia spectrum disorders. The current study aimed to evaluate the relative equivalence of two different programs designed to address the cognitive impairment associated with schizophrenia spectrum disorders, (CIRCuiTs and CCT) within a community mental health service. Both programs showed a large effect of time regarding the subjective assessment of cognition (SSTICS) and on the BACS the composite score, verbal memory, symbol coding, and token motor task. Interpretation of the results needs to be qualified by the limited sample size and power. In addition, the overall attrition rate was high and higher for the CCT group.

The previous meta-analysis has found various interventions to address the cognitive impairment of psychosis to be effective (3, 4, 18). The study by Kidd et al. also found no difference in restorative and compensatory approaches (6). Based on current evidence, including this study, services appear to have a choice of a range of effective approaches to delivering programs to address the cognitive impact of psychosis (3, 18). Decisions may vary based on the allocation of resources, cost, and fit of a program to the workforce or organizational context (19). Interventions that are too complex or expensive can be difficult to implement and maintain in routine mental healthcare.

This study explored the non-inferiority of CCT, which is an intervention requiring lower resources as it is freely available online compared with a computer-based program that requires infrastructure and often an annual license fee.

The extended impact of coronavirus disease 2019 (COVID-19) on group programs altered the mode of delivery of the study interventions, resulting in this study being halted prematurely. This limited the power of the study to generate conclusive results. In addition, clinically COVID-19 restrictions on the delivery of face-to-face care have highlighted the vulnerability of CR programs that require group or in-person delivery. Programs that can be delivered online, such as computer-based CR, may be necessary to deliver psychosocial interventions in the context of a pandemic.

The non-specific potential benefits over time of being involved in a structured program focused on cognition on improved self-efficacy and self-esteem were not evaluated in this study. It is known that people with schizophrenia can disengage from activities based on past experiences of failure and that the associated learned defeatist beliefs can impair functional recovery (20, 21). This may be a factor in the disappointing small or insignificant findings of CR on functional outcomes. CR may be a necessary but not sufficient intervention to begin to target functional improvement. This is supported in part by research where greater effect sizes are found for CR delivered in the context of rehabilitation programs (3). This study recruited from a general adult public community mental health service and did not control for other psychosocial interventions they may be receiving.

## Conclusion

This small study comparing two approaches to cognitive impairment in SSD, CCT and CIRCuiTS found no difference in effect based on the program used.

The main difference between these programs is that CCT does not require a computer and the manual can be accessed online for free. CCT uses less drill and practice than CIRCuiTS but this may not be as important as strategy-based learning, which is a common feature of the two approaches.

Due to the restrictions on psychosocial interventions due to COVID-19 restrictions, CIRCuiTS and other computer-based CR programs have the advantage that they can be delivered and supervised remotely online. The effectiveness of the online mode of delivery requires further study.

## Limitations

The main limitation of this study was the sample size due to premature cancellation of recruitment due to COVID-19 restrictions on therapy groups. In addition, the attrition rate was high. The lack of systematic recording of reasons for dropping out of the groups meant that important issues about the acceptability and feasibility of the respective programs could not be commented on. Groups were matched for total exposure to therapy (2 h per week for over 12 weeks) but not the frequency with CCT being offered in a 2-h once-a-week session and CIRCuiTS as two 1-h groups. Other psychosocial interventions delivered to participants in this study were not recorded or assessed as possible confounding variables.

Although there was a time effect for both conditions, without treatment as a usual group this may be due to regression to the mean or due to non-specific factors.

## Data Availability Statement

On completion of this study, the dataset will be made available from the corresponding author on reasonable request.

## Ethics Statement

The studies involving human participants were reviewed and approved by the Metro South Human Research Ethics Committee (AU/1/333D23) and the University of Queensland Human Research Ethics Committee (Clearance Number: 2018000962). All participation will be based on voluntary informed consent. The patients/participants provided their written informed consent to participate in this study.

## Author Contributions

FD, EN, VD, MW, and VG-J contributed to the design of the study. FD and VG-J drafted the protocol. FD and VD ran the CRT programs. VG-J and KN undertook the analysis. All authors have participated in the review and revision of the protocol manuscript and have approved the final manuscript to be published. The sponsor (Metro South Addiction and Mental Health Services, Woolloongabba) was not involved in the design and conduct of the study, the management, analysis, or interpretation of data, or the preparation, review, or approval of the manuscript for publication.

## Funding

In kind funding was provided by the Metro South Addiction and Mental Health Service but there was no additional or external funding provided for this research.

## Conflict of Interest

FD has received honorariums from Janssen, Lundbeck, and Seqirus for the delivery of lectures at clinician educational events. The remaining authors declare that the research was conducted in the absence of any commercial or financial relationships that could be construed as a potential conflict of interest.

## Publisher's Note

All claims expressed in this article are solely those of the authors and do not necessarily represent those of their affiliated organizations, or those of the publisher, the editors and the reviewers. Any product that may be evaluated in this article, or claim that may be made by its manufacturer, is not guaranteed or endorsed by the publisher.
